# Eyelid ptosis and dermatochalasis in obese patients with obstructive sleep apnoea: A large-scale propensity score-matched analysis

**DOI:** 10.1038/s41433-025-04164-8

**Published:** 2025-12-19

**Authors:** Aaron T. Zhao, Catherine Z. Shen, William R. Katowitz

**Affiliations:** 1 Perelman School of Medicine at the University of Pennsylvania, Philadelphia, PA USA; 2 Scheie Eye Institute, Department of Ophthalmology, Perelman School of Medicine at the University of Pennsylvania, Philadelphia, PA USA; 3 Division of Ophthalmology, Children’s Hospital of Philadelphia, Philadelphia, PA USA

**Keywords:** Epidemiology, Eyelid diseases, Respiratory tract diseases

## Abstract

**Background/objectives:**

While obstructive sleep apnoea (OSA) has been associated with various ocular manifestations, the relationship between OSA and eyelid disorders beyond floppy eyelid syndrome remains poorly characterised. This study investigated the association between OSA and eyelid ptosis and dermatochalasis in obese patients.

**Subjects/methods:**

A retrospective propensity score-matched cohort study was conducted using the TriNetX Research Network database (2015–2024). Obese patients with OSA confirmed by CPAP initiation (*n* = 315,223) were matched 1:1 with obese controls without OSA (*n* = 315,223) based on demographics and comorbidities. Primary outcomes included eyelid ptosis and dermatochalasis occurring after OSA diagnosis, analysed both as a composite outcome and as separate individual outcomes. Statistical analysis included risk ratios, odds ratios, number needed to harm (NNH), and time-to-event analysis with 95% confidence intervals.

**Results:**

Eyelid complications occurred in 7600 OSA patients (2.41%) versus 3890 controls (1.23%). OSA patients demonstrated significantly increased risk (risk ratio 1.95, 95% CI 1.88–2.03; odds ratio 1.98, 95% CI 1.90–2.06; NNH 119 patients, 95% CI 110–130; *p* < 0.0001). When analysed separately, ptosis occurred in 4760 OSA patients (1.51%) versus 2,112 controls (0.67%) with risk ratio 2.25 (95% CI 2.14–2.37, *p* < 0.0001). Dermatochalasis occurred in 4192 OSA patients (1.33%) versus 2207 controls (0.70%) with risk ratio 1.90 (95% CI 1.81–2.01, *p* < 0.0001).

**Conclusion:**

OSA is independently associated with a nearly two-fold increased risk of eyelid ptosis and dermatochalasis in obese patients, beyond the known association with floppy eyelid syndrome. These findings support enhanced ophthalmologic surveillance in OSA patients and highlight the importance of recognising ocular manifestations in sleep disorder populations.

## Introduction

Obstructive sleep apnoea (OSA), affecting approximately one billion individuals worldwide, is characterised by repetitive upper airway collapse during sleep, leading to intermittent hypoxia and sleep fragmentation [1, 2]. While primarily recognised as a respiratory disorder, OSA is increasingly understood to have significant systemic manifestations affecting multiple organ systems [3]. Ocular complications of OSA have gained attention in recent years, with established associations including floppy eyelid syndrome (FES), increased intraocular pressure, non-arteritic anterior ischemic optic neuropathy (NAION), and various anterior segment abnormalities [4, 5]. FES, characterised by eyelid laxity with easy eversion and chronic papillary conjunctivitis, is the most well-documented eyelid complication of OSA [6, 7].

Although FES is a well-recognised ocular complication of OSA, the association between OSA and other forms of eyelid ptosis and dermatochalasis remains poorly understood. Only one small clinical study has specifically examined ptosis and dermatochalasis prevalence in OSA patients [8]. Here, we conducted a large-scale, propensity score-matched cohort study to quantify the risk of eyelid ptosis and dermatochalasis in OSA patients compared to matched controls, focusing specifically on obese individuals, the population most affected by OSA [9].

## Materials and methods

We conducted a retrospective cohort study using the TriNetX Research Network, a federated health research network providing access to electronic health records from 66 healthcare organisations across the United States (2015–2024). Because this study used de-identified patient data, the University of Pennsylvania institutional review board approval was waived.

We identified obese patients with confirmed OSA defined by the presence of both diagnostic codes and treatment initiation. The OSA cohort inclusion criteria required: (1) diagnosis of obesity (ICD-10 E66.x) or high BMI documentation (ICD-10 Z68.41 or Z68.42 representing BMI 40.0–49.9 kg/m²); (2) OSA diagnosis (ICD-10 G47.33 or G47.30); and (3) CPAP initiation (CPT 94660) to ensure treatment-confirmed OSA rather than suspected disease. The control cohort consisted of obese patients without any OSA diagnosis throughout the observation period, requiring obesity diagnosis codes (ICD-10 E66.x or Z68.41–Z68.42) but excluding those with any sleep apnoea diagnosis codes and requiring age ≥18 years. Both cohorts excluded patients with congenital ptosis (ICD-10 Q10.0), FES or other specified eyelid disorders (ICD-10 H02.89) present before the index date to focus on acquired eyelid ptosis and dermatochalasis potentially related to OSA.

Propensity score matching between OSA patients and controls (1:1) was performed using a greedy nearest-neighbour algorithm without replacement. Matching variables included demographics (age, sex, race, ethnicity), comorbidities (hypertension, diabetes, ischemic heart disease, lifestyle problems, sleep disorders), medications (benzodiazepines, sedatives/hypnotics), and BMI measurements. The primary outcome was development of eyelid ptosis (ICD-10 H02.4) or dermatochalasis (ICD-10 H02.83) occurring after the index date. Outcomes were analysed both as a composite endpoint (patients with either ptosis or dermatochalasis or both conditions) and as separate individual outcomes to assess condition-specific associations with OSA. The index event for both cohorts was defined as the first diagnosis of obesity (BMI ≥ 30 kg/m²). Outcomes were analysed starting 1 day after the index event through the end of available follow-up, with patients excluded if their index event occurred more than 20 years prior to analysis.

Statistical analysis included risk ratios, odds ratios, number needed to harm (NNH) with 95% confidence intervals calculated as the reciprocal of absolute risk differences, and 95% confidence intervals for both the composite eyelid outcome and individual outcomes. Standardised mean differences were calculated to assess covariate balance after propensity score matching. Time-to-event analysis was performed using Kaplan-Meier survival curves with log-rank tests and Cox proportional hazards models. Follow-up duration between groups was compared using the Mann-Whitney U test. Patients were censored at their last recorded healthcare encounter. All analyses were performed using the TriNetX platform, with statistical significance set at *p* < 0.05.

## Results

Before propensity score matching, the initial cohorts included 325,716 OSA patients and 5,323,932 controls. After excluding 10,424 OSA patients and 134,210 controls whose index events occurred more than 20 years prior, the remaining eligible populations underwent propensity score matching. The reduction in controls from the initial pool to the final matched cohort (315,223) occurred primary during the 1:1 propensity score matching process to ensure optimal balance of measured confounders. Final propensity score matching yielded 315,223 OSA patients and 315,223 matched controls for final analysis.

The matched cohorts were well-balanced across demographic and clinical characteristics (Table 1). Mean age was 65.6 years in both groups, with 55.7% male in the OSA group and 55.4% in controls. Racial distribution showed 73.1% White patients in both cohorts, and 70.3% were non-Hispanic. Comorbidity prevalence was similar between groups: diabetes mellitus (28.7% vs. 28.8%), essential hypertension (48.6% vs. 48.5%), and chronic ischemic heart disease (18.1% vs. 17.8%). Sleep-related medications showed minimal differences, with benzodiazepines prescribed in 34.0% of OSA patients versus 33.7% of controls, and sedatives/hypnotics in 13.1% versus 12.6%. All standardised mean differences were <0.02, indicating excellent matching. Mean BMI was 37.9 ± 7.7 kg/m² in OSA patients and 36.4 ± 7.1 kg/m² in controls. Follow-up duration was significantly longer in the OSA cohort (*p* < 0.0001), with mean follow-up of 2088 days (5.7 years) compared to 1429 days (3.9 years) in controls. Median follow-up was 1923 days in OSA patients and 1049 days in controls.

**Table 1:** Baseline Characteristics of Obese Patients with and without OSA After Propensity Score Matching.

Characteristic	OSA Patients (*n* = 315,223)	Controls (*n* = 315,223)	Std Diff
Demographics			
Age, mean ± SD (years)	65.6 ± 14.4	65.6 ± 14.2	0.018
Male, n (%)	175,584 (55.7%)	174,692 (55.4%)	0.006
White race, n (%)	230,386 (73.1%)	230,526 (73.2%)	0.003
Not Hispanic or Latino, n (%)	221,474 (70.3%)	221,582 (70.3%)	0.001
Comorbidities			
Essential hypertension, n (%)	153,089 (48.6%)	153,030 (48.5%)	<0.001
Diabetes mellitus, n (%)	90,578 (28.7%)	90,781 (28.8%)	0.001
Chronic ischemic heart disease, n (%)	56,899 (18.1%)	56,209 (17.8%)	0.006
Problems related to lifestyle, n (%)	12,345 (3.9%)	11,367 (3.6%)	0.016
Sleep disorders (non-OSA), n (%)	6,723 (2.1%)	5,754 (1.8%)	0.022
BMI Categories			
BMI 30-35 kg/m², n (%)	78,035 (24.8%)	79,026 (25.1%)	0.007
BMI 35-40 kg/m², n (%)	73,818 (23.4%)	73,894 (23.4%)	0.001
BMI 40-100 kg/m², n (%)	69,093 (21.9%)	68,868 (21.8%)	0.001
Medications			
Benzodiazepines, n (%)	107,179 (34.0%)	106,207 (33.7%)	0.007
Sedatives/hypnotics, n (%)	41,251 (13.1%)	39,871 (12.6%)	0.013

Eyelid ptosis and dermatochalasis (excluding FES) occurred in 7600 of 315,223 OSA patients (2.41%) compared to 3890 of 315,223 controls (1.23%). The absolute risk difference was 1.18% (95% CI 1.11–1.24%, *p* < 0.0001). OSA patients demonstrated a risk ratio of 1.954 (95% CI 1.88–2.03) and odds ratio of 1.977 (95% CI 1.902–2.056), both reaching statistical significance (*p* < 0.0001) (Table 2). The NNH was 85 patients (95% CI 80–90), indicating that for every 85 obese patients with OSA followed over the study period, one additional case of eyelid complications would be expected compared to matched controls. When analysed as separate outcomes, both ptosis and dermatochalasis showed significant associations with OSA (Table 2). Ptosis occurred in 4760 of 315,223 OSA patients (1.51%) compared to 2112 of 315,223 controls (0.67%), yielding an odds ratio of 2.27 (95% CI 2.15–2.39, *p* < 0.0001), risk ratio of 2.25 (95% CI 2.14–2.37, *p* < 0.0001), and NNH of 119 patients (95% CI 110–130) (Table 3a). Dermatochalasis occurred in 4,192 of 315,223 OSA patients (1.33%) compared to 2207 of 315,223 controls (0.70%), resulting in an odds ratio of 1.91 (95% CI 1.82–2.02, *p* < 0.0001), risk ratio of 1.90 (95% CI 1.81–2.01, *p* < 0.0001), and NNH of 159 patients (95% CI 145–175) (Table 3b).

**Table 2:** Composite outcome analysis: ptosis and dermatochalasis.

Analysis Type	Measure	OSA Patients	Controls	Statistic	95% CI	*P*-value
**Event Rates**	Patients with outcome, n (%)	7600 (2.41%)	3890 (1.23%)	—	—	—
**Risk Analysis**	Risk difference	—	—	1.18%	1.11-1.24%	<0.0001
	Risk ratio	—	—	1.95	1.88-2.03%	<0.0001
	Odds ratio	—	—	1.98	1.90-2.06%	<0.0001
**Survival Analysis**	Event-free survival at study end	90.64%	93.94%	—	—	—
	Hazard ratio	—	—	1.39	1.33–1.44	<0.0001
	Log-rank test	—	—	χ² = 275.126	df = 1	<0.0001

**Table 3:** a Individual Outcome Analysis: Ptosis; b Individual Outcome Analysis: Dermatochalasis.

Analysis Type	Measure	OSA Patients (*n* = 315,223)	Controls (*n* = 315,223)	Statistic	95% CI	*P*-value
**a Individual Outcome Analysis: Ptosis**						
**Event Rates**	Patients with outcome, n (%)	4760 (1.51%)	2112 (0.67%)	—	—	—
**Risk Analysis**	Risk difference	—	—	0.84%	0.77%–0.91%	<0.0001
	Risk ratio	—	—	2.25	2.14–2.37	<0.0001
	Odds ratio	—	—	2.27	2.15–2.39	<0.0001
**Survival Analysis**	Event-free survival at study end	94.38%	96.20%	—	—	—
	Hazard ratio	—	—	1.60	1.52–1.68	<0.0001
	Log-rank test	—	—	χ² = 318.603	df = 1	<0.0001
**b Individual Outcome Analysis: Dermatochalasis**						
**Event Rates**	Patients with outcome, n (%)	4192 (1.33%)	2207 (0.70%)	—	—	—
**Risk Analysis**	Risk difference	—	—	0.63%	0.57%–0.69%	<0.0001
	Risk ratio	—	—	1.90	1.81–2.01	<0.0001
	Odds ratio	—	—	1.91	1.82–2.02	<0.0001
**Survival Analysis**	Event-free survival at study end	95.20%	96.16%	—	—	—
	Hazard ratio	—	—	1.33	1.26–1.40	<0.0001
	Log-rank test	—	—	χ² = 115.917	df = 1	<0.0001

Kaplan-Meier survival analysis showed that 90.6% of OSA patients remained free of eyelid complications compared to 93.9% of controls at the end of the observation period (maximum follow-up approximately 20 years from index event) (Table 2). Cox proportional hazards analysis yielded a hazard ratio of 1.39 (95% CI 1.33-1.44, *p* < 0.0001). The log-rank test showed χ² = 275.126, df = 1, *p* < 0.0001. Neither group reached median survival time during the observation period. Individual outcome survival analyses (Tables 3a and 3b) showed that for ptosis alone, 94.38% of OSA patients remained ptosis-free compared to 96.20% of controls, with a hazard ratio of 1.60 (95% CI 1.52–1.68, *p* < 0.0001). For dermatochalasis alone, 95.20% of OSA patients remained dermatochalasis-free compared to 96.16% of controls, with a hazard ratio of 1.33 (95% CI 1.26–1.40, *p* < 0.0001).

## Discussion

This large-scale, propensity score-matched analysis provides robust population-level evidence that OSA is independently associated with significantly increased risk of eyelid ptosis and dermatochalasis beyond the well-established association with FES. [6, 7] The nearly two-fold increased risk (RR 1.95) and 39% increased hazard (HR 1.39) in the composite analysis, with NNH of 85 patients, represent clinically meaningful effect sizes that persist throughout extended follow-up periods. The individual outcome effects of ptosis (RR 2.25, HR 1.60, NNH 119) and dermatochalasis (RR 1.90, HR 1.33, NNH 159) demonstrate robust associations for both conditions.

Several mechanisms may explain the association between OSA and eyelid disorders. Chronic intermittent hypoxia, the hallmark of OSA, triggers a cascade of pathophysiological changes including increased oxidative stress, inflammation, and endothelial dysfunction [10–13]. These processes may accelerate aging of periocular tissues and compromise the structural integrity of eyelid support mechanisms. Elevated inflammatory markers commonly seen in OSA patients, including tumour necrosis factor-α, interleukin-6, and C-reactive protein, may promote collagen degradation and tissue remodelling that predispose to ptosis and dermatochalasis [13, 14].

The mechanical aspects of OSA may also contribute to eyelid changes. Repeated episodes of upper airway obstruction create negative intrathoracic pressure and increased venous pressure, potentially affecting periocular venous drainage and contributing to tissue oedema and stretching. [15] OSA is also associated with accelerated aging processes and premature development of age-related conditions. [16–19] The development of ptosis and dermatochalasis at younger ages in OSA patients would be consistent with this pattern of accelerated senescence affecting multiple organ systems.

These findings have important practical implications for clinical care. The NNH calculations indicate that for every 85–159 obese OSA patients followed clinically, one additional case of eyelid pathology would be expected compared to similar patients without OSA. These findings have several important clinical implications. First, they support the need for enhanced ophthalmologic surveillance in OSA patients. While routine eye care is already recommended for OSA patients due to associations with glaucoma, FES, and other ocular conditions, our results suggest that eyelid examination should specifically include assessment for ptosis and dermatochalasis beyond the traditional focus on FES [20, 21]. Early detection of these conditions could prompt timely intervention and may serve as additional markers for OSA severity or treatment adequacy. However, prospective studies are needed to establish these relationships.

Second, ophthalmologists evaluating patients with ptosis or dermatochalasis, particularly in overweight individuals, should maintain awareness of potential underlying sleep disorders. Unlike FES, which has pathognomonic features that readily suggest OSA, ptosis and dermatochalasis may appear similar to age-related changes, potentially masking their association with sleep disorders [22]. This represents an opportunity for ophthalmologists to contribute to the identification of undiagnosed OSA, which remains underrecognised in the general population, with studies suggesting that up to 80% of moderate to severe cases remain undiagnosed [23, 24].

Third, the substantial effect sizes observed suggest that OSA may be a modifiable risk factor for eyelid disorders. Effective OSA treatment with continuous positive airway pressure therapy or other interventions might potentially reduce the risk of developing these complications, though this hypothesis would require prospective validation. Additionally, the association between OSA and eyelid pathology has important surgical implications. A recent study by Nirmalan et al. demonstrated that OSA patients undergoing ptosis repair surgery had significantly higher failure rates compared to non-OSA patients, with a 1.7-fold increased risk of requiring revision surgery [25]. This finding supports our results and suggests that OSA-related tissue changes may compromise surgical outcomes, further emphasising the importance of identifying this association preoperatively.

This investigation has several notable strengths. The large sample size of over 630,000 patients provides robust statistical power and precise effect estimates. The propensity score matching successfully balanced measured confounders, with all standardised mean differences <0.02, indicating excellent balance as recommended for large datasets where traditional p-values may be misleading due to sample size [26]. The focus on obese patients reduced body mass index as a major confounding variable while studying the population most clinically relevant to OSA management. The requirement for CPAP initiation in the OSA group ensures confirmed rather than suspected disease, strengthening the exposure definition. The use of the TriNetX Research Network provides geographic and demographic diversity across several healthcare organisations, enhancing the generalisability of findings. The extended follow-up period averaging over 5 years in OSA patients allows for adequate time for outcome development and demonstrates the persistence of increased risk over time.

Several limitations should be acknowledged. The differential follow-up duration between cohorts (5.7 vs. 3.9 years, *p* < 0.0001) introduces potential surveillance bias, where longer observation periods in OSA patients could increase detection of eyelid complications. While our time-to-event analyses using Cox proportional hazards models help mitigate this concern by accounting for differential observation times, this limitation could contribute to overestimation of the association magnitude. Moreover, the TriNetX database lacks information on OSA severity (apnoea-hypopnea index), treatment adherence, or CPAP efficacy, preventing assessment of dose-response relationships or the potential protective effects of successful treatment. Similarly, lifestyle factors such as smoking history, sun exposure, and genetic predisposition are not reliably available, limiting our ability to control for these potential confounders. The reliance on ICD-10 diagnostic codes may result in outcome misclassification; however, ptosis and dermatochalasis are typically well-documented conditions requiring clinical examination for diagnosis. The potential for undiagnosed OSA in the control group would bias results toward the null hypothesis, suggesting our findings may underestimate the true association. The study population is limited to obese individuals with healthcare access, potentially limiting generalisability to non-obese populations or those with limited healthcare access.

The temporal relationship between OSA and eyelid complications, while appropriate for the study design, does not definitively establish causation. Reverse causation is unlikely given the biological plausibility of OSA preceding eyelid changes, but residual confounding cannot be entirely excluded.

Our choice of 1:1 matching was designed to optimise covariate balance while maintaining adequate statistical power. Although higher matching ratios (e.g., 1:3 or 1:5) were feasible given our large control pool, 1:1 matching provides the best balance between statistical efficiency and covariate matching quality, particularly important given the numerous potential confounding variables in OSA research. This approach substantially reduced our analytical sample size but ensured excellent covariate balance as evidenced by the standardised mean differences <0.02.

These findings establish a foundation for several important research directions. Prospective studies examining the relationship between OSA severity, treatment adequacy, and eyelid complication development would provide insights into potential preventive strategies. Investigation of inflammatory biomarkers and tissue changes in OSA patients with and without eyelid disorders could elucidate underlying mechanisms. Clinical trials examining whether effective OSA treatment reduces eyelid complication risk would have significant therapeutic implications. Development of standardised screening protocols for eyelid disorders in OSA patients and sleep disorder assessment in ophthalmology patients could improve coordinated care and early detection of both conditions.

## Conclusions

This large-scale, propensity score-matched analysis demonstrates that OSA is independently associated with a nearly two-fold increased risk of eyelid ptosis and dermatochalasis in obese patients, distinct from the well-established association with FES. Individual outcome analyses reveal that both ptosis and dermatochalasis demonstrate robust individual associations with OSA, with ptosis showing a somewhat stronger effect. The clinical impact, demonstrated through NNH calculations, indicates meaningful population-level effects that warrant consideration in routine clinical care. These findings support enhanced ophthalmologic surveillance in OSA patients and emphasise the importance of multidisciplinary awareness of this association. Future research should focus on mechanistic understanding, severity relationships, and potential therapeutic interventions to reduce eyelid complication risk in this vulnerable population.

## Summary

### What was known before

- OSA has established associations with various ocular complications including floppy eyelid syndrome, increased intraocular pressure, and non-arteritic anterior ischemic optic neuropathy.
- Only one small clinical study had previously examined the prevalence of ptosis and dermatochalasis in OSA patients, leaving this association poorly characterised.

### What this study adds

- Large-scale population-level evidence demonstrating that OSA is independently associated with nearly two-fold increased risk of eyelid ptosis and dermatochalasis beyond floppy eyelid syndrome.
- Propensity score-matched analysis of over 630,000 patients revealed specific odds ratios of 2.27 for ptosis and 1.91 for dermatochalasis in OSA patients compared to controls.
- Establishes that both ptosis and dermatochalasis demonstrate robust individual associations with OSA, with ptosis showing a somewhat stronger effect than dermatochalasis.

## Data Availability

The data that support the findings of this study are available from TriNetX but restrictions apply to the availability of these data and so are not publicly available.
